# Rare hereditary cause of chronic pancreatitis in a young male: SPINK1 mutation

**DOI:** 10.11604/pamj.2017.28.110.13854

**Published:** 2017-10-04

**Authors:** Janaki Patel, Arina Madan, Amanda Gammon, Michael Sossenheimer, Niloy Jewel Samadder

**Affiliations:** 1 Division of Gastroenterology, University of Utah, Salt Lake City, Utah, USA; 2 University of Birmingham Medical School, Edgbaston, Birmingham, B15 2TT, UK; 3 Huntsman Cancer Institute, University of Utah, Salt Lake City, Utah, USA; 4 Division of Gastroenterology,Hepatology and Nutrition, University of Utah, Salt Lake City, Utah, USA

**Keywords:** Hereditary pancreatitis, SPINK1 mutation, atypical presentation, pancreatic malignancy

## Abstract

Hereditary chronic pancreatitis associated with a mutation in the serine protease inhibitor, Kazal Type-1 (SPINK-1 gene) is extremely rare. The SPINK1 mutation results in trypsinogen activation which predisposes to chronic pancreatitis predominately when combined with CFTR gene mutations. It presents as either chronic or recurrent acute pancreatitis. Symptom control and management of complications is important. Active surveillance with cross-sectional imaging for pancreatic malignancy in individuals with hereditary pancreatitis is advocated due to individuals being high risk. We present an unusual case of a young male who initially presented with renal colic and was incidentally diagnosed with severe chronic pancreatitis on abdominal imaging, with genetic testing confirming a homozygous SPINK1 mutation.

## Introduction

Mutation in the serine protease inhibitor, Kazal type-1 (SPINK1 gene) increases the chance of an individual developing chronic pancreatitis 12-fold [1]. Inheritance is autosomal recessive due to the need for mutations in both copies of the SPINK1 gene, thus one mutant copy is inherited from each parent who are unaffected carriers. Mutations in the SPINK1 gene lead to premature trypsinogen activation and resultant pancreatitis [2-4]. SPINK mutation associated pancreatitis is extremely rare, with under 1% of carriers proceeding to develop pancreatitis [5]. Other commonly identified genes whose mutations are associated with chronic pancreatitis include the cationic trypsinogen gene (PRSS1) and Cystic Fibrosis Transmembrane Conductance Regulator gene (CFTR), with the later gene being the most common genetic variant to co-exist with SPINK1 mutations [2, 3].

## Patient and observation

**Figure 1:**
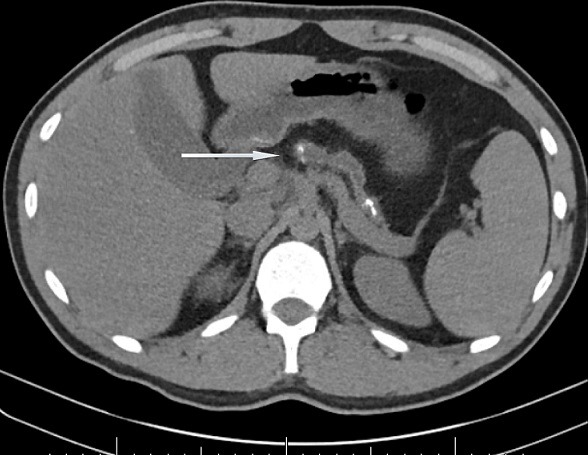
CT abdomen showing pancreatic atrophy with multiple sub-centimeter calcifications consistent with severe chronic pancreatitis: arrow points to dilated pancreatic duct with stones in it.

**Figure 2:**
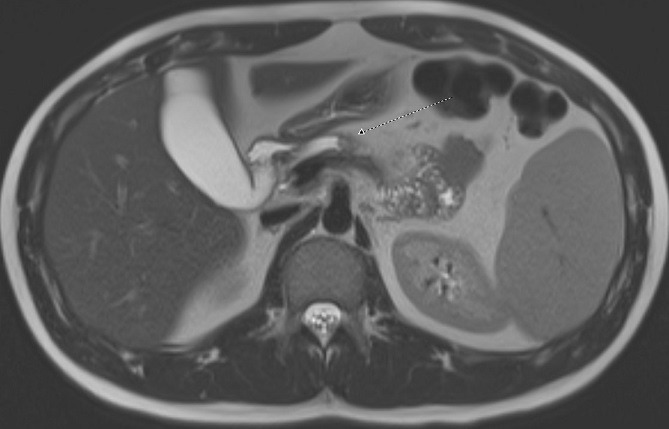
Magnetic resonance cholangiopancreatography (MRCP) confirming severe parenchymal atrophy and pancreatic ductal stones with ductal dilation to 9mm: arrow points to dilated pancreatic duct with stones in it

A 21-year-old male was referred to the gastroenterology clinic for incidental findings of severe chronic pancreatitis on cross-sectional imaging but no associated symptoms of pancreatitis. He had presented to the emergency department for abdominal pain consistent with prior episodes of renal colic. Computed tomography scan confirmed the presence of renal calculi that passed with conservative management. An incidental finding on the imaging showed marked pancreatic atrophy with multiple sub-centimeter calcifications consistent with severe chronic pancreatitis (Figure 1). Remarkably, he did not report having any symptoms associated with pancreatic insufficiency including epigastric pain, vomiting, steatorrhea and weight loss. He denied having any prior episodes of acute pancreatitis and had no history of alcohol use. His laboratory tests were all within normal range, including complete blood count, liver function tests, amylase and lipase. His family history was significant as his father had an isolated episode of pancreatitis which required abdominal surgery. Magnetic resonance cholangiopancreatography (MRCP) showed severe parenchymal atrophy and pancreatic ductal stones with ductal dilation to 9mm (Figure 2). Due to his young age and atypical presentation, he was referred for genetic testing and counselling. He underwent genetic testing for various genes known to cause hereditary pancreatitis: CASR, CFTR, CTRC, PRSS1, SPINK1. He tested positive for homozygous variant of SPINK1 (N34S) mutation. Reflex testing of at-risk relatives confirmed that both of his parents were heterozygous carriers of the same SPINK1 (N34S) mutation and a review of their extended pedigree revealed that they were distant cousins. Given his asymptomatic clinical course, he remains under close follow-up without requiring any specific treatment for pancreatic insufficiency. Due to the severity of pancreatitis noted on imaging and long expected lifespan, he was counselled about the increased risk of pancreatic malignancy and is undergoing regular cross-sectional imaging for the detection and prevention of cancer.

## Discussion

Hereditary pancreatitis is a rare cause of chronic pancreatitis, with mutations in PRSS1, SPINK1, and CFTR genes being the most common genetic causes. Hereditary pancreatitis significantly increases the risk of pancreatic malignancy [2-5]. The most common cause of hereditary pancreatitis is an autosomal dominant form caused by mutations in the PRSS1 gene which account for up to 80% of cases [4]. While mutations in the PRSS1 cause chronic pancreatitis on their own, the SPINK1 mutation is instead likely to act as a disease modifier, lowering the threshold for an individual developing pancreatitis from other genetic factors such as the CFTR mutations and environmental factors. Patients with heterozygous CFTR mutations who develop chronic pancreatitis often have coexisting SPINK1 mutations which indicates there is an interaction between these genes. A study by Schneider and colleagues found that coinheritance of cystic fibrosis causing CFTR variants with SPINK1 variants significantly increases the risk of idiopathic chronic pancreatitis, with 36% of the 53 patients with cystic fibrosis having a SPINK1 mutation [5]. While up to 2% of the *general population* carry SPINK1 mutations, the actual number of individuals with SPINK1 associated pancreatitis is extremely rare, with less than 1% of carriers going on to developing pancreatitis [6]. Despite this, carrying the SPINK1 mutation increases an individual's risk of developing chronic pancreatitis 12-fold. The prevalence of SPINK1 mutations in *patients* with idiopathic chronic pancreatitis has been reported as between 16-23%, with a case series reporting that SPINK1 mutations were 16.9% more common in patients with chronic and recurrent acute pancreatitis than controls [1, 5, 7].

The most common variant of the SPINK1 mutation is the N34S variant. However, there are also reports of another variant called the IVS3+2T > C variant, which is present in individuals from China, Korea and Japan. A study of 86 Japanese patients with chronic pancreatitis and 527 healthy volunteers found that both the N34S and IVS3+2T > C variant were commoner in the patients compared to healthy controls, 12.9% vs 0.37% and 8.6% vs 0%, respectively [8]. SPINK1 encodes a pancreatitis secretory trypsin inhibitor which is released by pancreatic acinar cells when there is inflammation. Mutation in the SPINK1 gene leads to trypsin being uninhibited which increases the risk of pancreatitis [9]. Most patients have heterozygous SPINK1 mutations leading to complex inheritance patterns, although SPINK1 variants have also been associated with autosomal recessive familiar pancreatitis, alcoholic pancreatitis and tropical pancreatitis [10]. Clinically, hereditary pancreatitis presents as either chronic pancreatitis or recurrent acute pancreatitis and normally presents before the age of 20. It classically presents with epigastric abdominal pain radiating to the back, steatorrhea secondary to malabsorption and pancreatic diabetes due to islet cell damage [11]. The diagnosis of hereditary pancreatitis is based on a combination of factors including genetic testing, history, physical examination, laboratory results and radiographic imaging [4]. Treatment of hereditary pancreatitis is the same as the management of other causes of chronic pancreatitis and primarily involves administration of analgesia, pancreatic enzymes, fat-soluble vitamins, sometimes insulin and avoidance of alcohol and tobacco, as well as a low-fat diet. In severe cases, pancreatectomy with islet cell transplantation can be considered [12-14]. Lifetime risk of developing pancreatic cancer in hereditary chronic pancreatitis is approximately 40%. Screening with EUS or MRCP is recommended in hereditary chronic pancreatitis, however, exact interval is undefined due to its cost effectiveness and sensitivity of these tests [15].

## Conclusion

Our case of SPINK mutation associated pancreatitis is extremely rare. This case is made even more unique by the atypical and asymptomatic presentation of the severe chronic pancreatitis, discovered incidentally on imaging for renal colic. This case highlights the importance that clinicians should have a high index of suspicion that hereditary pancreatitis may be the underlying cause of chronic pancreatitis in younger patients. It is vital that patients who test positive for hereditary pancreatitis, including the SPINK1 mutation undergo active surveillance for pancreatic malignancy due to them being in a high-risk group. This will allow early treatment and ensure the best prognosis in these rare cases of hereditary pancreatitis.

## Competing interests

The authors declare no competing interests.

## References

[1] Teich N, Bauer N, Mossner J, Keim V (2002). Mutational screening of patients with nonalcoholic chronic pancreatitis: identification of further trypsinogen variants. Am J Gastroenterol..

[2] Whitcomb DC, Gorry MC, Preston RA, Furey W, Sossenheimer MJ, Ulrich CD (1996). Hereditary pancreatitis is caused by a mutation in the cationic trypsinogen gene. Nat Genet..

[3] Whitcomb DC, Preston RA, Aston CE, Sossenheimer MJ, Barua PS, Zhang Y (1996). A gene for hereditary pancreatitis maps to chromosome 7q35. Gastroenterology..

[4] Sossenheimer MJ, Aston CE, Preston RA, Gates LK Jr, Ulrich CD, Martin SP (1997). Clinical characteristics of hereditary pancreatitis in a large family, based on high-risk haplotype: the midwest multicenter pancreatic study group (MMPSG). Am J Gastroenterol..

[5] Schneider A, Larusch J, Sun X, Aloe A, Lamb J, Hawes R (2011). Combined bicarbonate conductance-impairing variants in CFTR and SPINK1 variants are associated with chronic pancreatitis in patients without cystic fibrosis. Gastroenterology..

[6] Pfutzer RH, Barmada MM, Brunskill AP, Finch R, Hart PS, Neoptolemos J (2000). SPINK1/PSTI polymorphisms act as disease modifiers in familial and idiopathic chronic pancreatitis. Gastroenterology..

[7] Witt H, Luck W, Hennies HC, Classen M, Kage A, Lass U (2000). Mutations in the gene encoding the serine protease inhibitor, kazal type 1 are associated with chronic pancreatitis. Nat Genet..

[8] Shimosegawa T, Kume K, Masamune A (2008). SPINK1, ADH2 and ALDH2 gene variants and alcoholic chronic pancreatitis in japan. J Gastroenterol Hepatol..

[9] DiMagno EP, DiMagno MJ (2016). Chronic pancreatitis: Landmark papers, management decisions and future. Pancreas.

[10] Aoun E, Chang CC, Greer JB, Papachristou GI, Barmada MM, Whitcomb DC (2008). Pathways to injury in chronic pancreatitis: decoding the role of the high-risk SPINK1 N34S haplotype using meta-analysis. PLoS One.

[11] Patel MR, Eppolito AL, Willingham FF (2013). Hereditary pancreatitis for the endoscopist. Therap Adv Gastroenterol..

[12] Forsmark CE (2013). Management of chronic pancreatitis. Gastroenterology.

[13] Chinnakotla S, Bellin MD, Schwarzenberg SJ, Radosevich DM, Cook M, Dunn TB (2014). Total pancreatectomy and islet autotransplantation in children for chronic pancreatitis: indication, surgical techniques, postoperative management and long-term outcomes. Ann Surg..

[14] Chinnakotla S, Beilman GJ, Dunn TB, Bellin MD, Freeman ML, Radosevich DM (2015). Factors predicting outcomes after a total pancreatectomy and islet autotransplantation lessons learned from over 500 cases. Ann Surg..

[15] Greer JB, Brand RE (2007). Screening for pancreatic cancer: Current evidence and future directions. Gastroenterol Hepatol (NY).

